# Efficacy and safety of electroacupuncture on treating mild to moderate first-episode depression: a study protocol for a randomized controlled trial

**DOI:** 10.3389/fpsyt.2025.1521859

**Published:** 2025-02-26

**Authors:** Jingyu Xia, Minghui Jiang, Xuan Yin, Zuqing Wang, Feng Li, Haiyan Wei, Chuanyun Jin, Yanmei Hu, Jianhua Chen, Shifen Xu

**Affiliations:** ^1^ Department of Acupuncture and Moxibustion, Shanghai Municipal Hospital of Traditional Chinese Medicine, Shanghai University of Traditional Chinese Medicine, Shanghai, China; ^2^ International Education College, Shanghai University of Traditional Chinese Medicine, Shanghai, China; ^3^ Department of Acupuncture and Moxibustion, Shanghai Research Institute of Acupuncture and Meridian, Shanghai, China; ^4^ Department of Chinese Traditional Medicine, Shanghai Pudong New District Nanhui Mental Health Center, Shanghai, China; ^5^ Department of Sleep Disorders, Shanghai Pudong New District Nanhui Mental Health Center, Shanghai, China; ^6^ Department of Acupuncture and Moxibustion, Shanghai Xuhui District Central Hospital, Shanghai, China; ^7^ Shanghai Mental Health Center, Shanghai Jiao Tong University School of Medicine, Shanghai Key Laboratory of Psychotic Disorders, Shanghai, China

**Keywords:** electroacupuncture, depression, protocol, randomized controlled trial, non-inferiority

## Abstract

**Introduction:**

Only 30%-40% of patients with first-episode depression recover after taking antidepressants. Acupuncture is a clinically recognized treatment for depression, but its effect on first-episode depression remains unknown. This randomized controlled trial is designed to investigate the efficacy and safety of electroacupuncture (EA) compared with escitalopram (ESC) in treating patients with mild to moderate first-episode depression.

**Methods and analysis:**

This is a multi-site, single-blind, randomized controlled trial with two parallel arms. A total of 204 eligible patients will be randomly allocated to two groups: the EA group (receiving EA treatment with placebo drugs) and the ESC group (receiving escitalopram and sham acupuncture treatment). Treatment will last 12 weeks, with 3 sessions per week for the first 8 weeks, decreasing to 2 per week for the remaining 4 weeks. The primary outcome will be the score of the 17-item Hamilton Depression Rating Scale (HAMD-17), and the secondary outcomes will include depression recovery rate, depression remission rate, Patient Health Questionnaire-9 (PHQ-9), 36-Item Short Form Survey Instrument (SF-36), and the dose and frequency of ESC. The Treatment Emergent Symptom Scale (TESS) will be used to assess all adverse effects. Full details of the statistical analysis plan for the primary and secondary outcomes will be described in this article.

## Introduction

Depression is an emotional disorder that endangers human physical and mental health and is characterized by persistent sadness and/or loss of pleasure in previously enjoyable activities. Approximately 300 million people worldwide suffer from depression, which has become a major cause of disability and significantly contributes to the global disease burden (1, 2). Depression is the most common mood disorder in China, with a lifetime prevalence of 3.4% and a prevalence of 2.1% at twelve months (3).Selective serotonin reuptake inhibitors (SSRIs) are currently the first-line agents for treating depression. However, due to the complex etiology, only 30%-40% of patients with first-episode depression achieve clinical recovery after treatment with antidepressants alone (4, 5). While SSRIs can enhance the effectiveness in treating depression, it is important not to disregard their known adverse effects, including nausea, vomiting, tolerance, addiction, excessive sedation, and neurological damage (6, 7). Thus, it is urgent to develop an effective and safe treatment for patients with mild to moderate first-episode depression, especially for those potential non-responders.

In contrast to therapy with SSRIs alone, manual acupuncture (MA) as an add-on therapy for SSRIs can effectively improve the Hamilton Depression Scale (HAMD-17) scores and depression self-rating scale (SDS) scores to a greater extent (8). A randomized controlled trial has also shown that the efficacy of electroacupuncture (EA), a combination of acupuncture and low-pulse electrical stimulation, is not less than that of antidepressants (8, 9). However, evidence remains insufficient in relation to the comparison between the efficacy of EA and SSRIs, and thus the question of whether EA is not inferior to SSRIs in treating first-episode depression remains inconclusive.

We therefore designed this patient-blinded, randomized, and placebo-controlled trial with a sufficient observation period in Shanghai, China. The main objective of this trial is to explore whether EA is a viable alternative to SSRIs for patients diagnosed with mild to moderate first-episode depression.

## Methods and analysis

### Hypothesis

We aim to provide definitive proof to validate the hypothesis that EA is not inferior to escitalopram (ESC) in the treatment of mild to moderate first-episode depression.

### Design

This is a multi-center, patient-assessor-blinded, randomized, and controlled trial designed to evaluate the efficacy and safety of EA treatment in patients with mild to moderate first-episode depression and to compare the therapeutic effect between EA and ESC.

**Figure 1:**
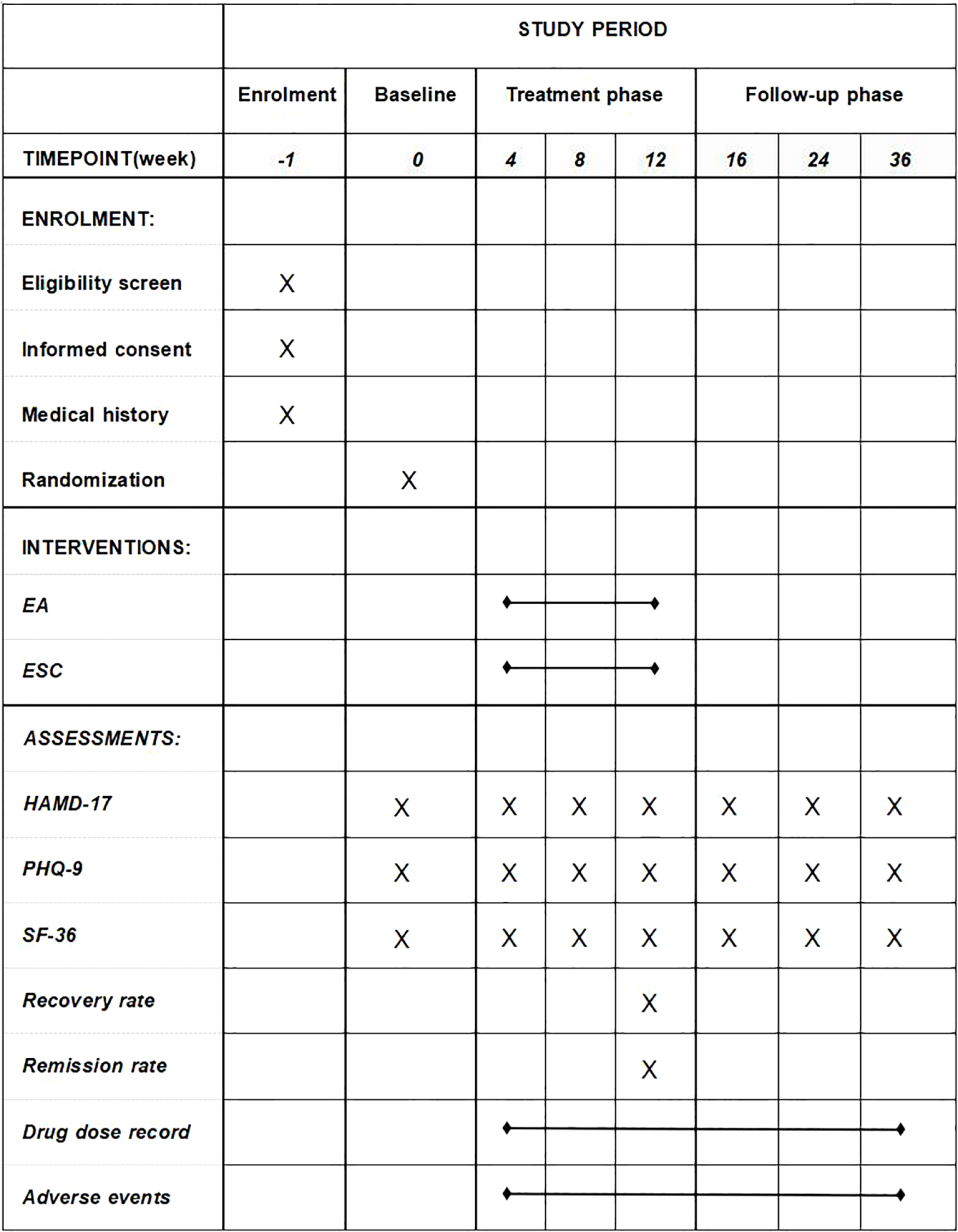
Schedule of enrolment, interventions, and assessments. EA, electroacupuncture; ESC, escitalopram; HAMD-17, 17-item Hamilton Rating Scale for Depression; PHQ-9, The Patient Health Questionnaire-9; SF-36, 36-Item Short Form Survey Instrument.

**Figure 2:**
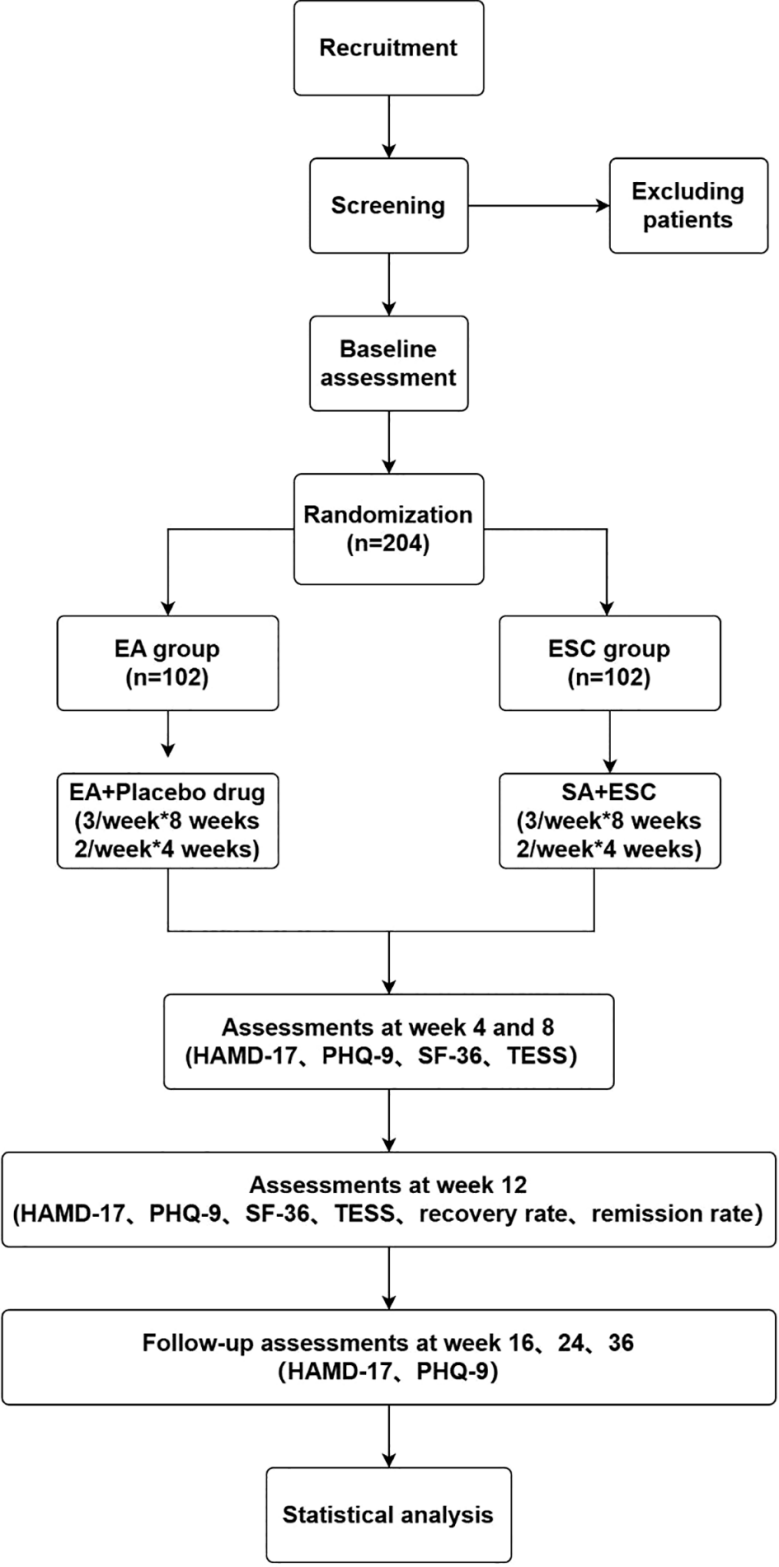
Flowchart of the study. EA, electroacupuncture; SA, sham acupuncture; ESC, escitalopram; HAMD-17, 17-item Hamilton Rating Scale for Depression; PHQ-9, The Patient Health Questionnaire-9; SF-36, 36-Item Short Form Survey Instrument; TESS, Treatment Emergent Symptom Scale.

We will conduct this study at 4 centers, including Shanghai Municipal Hospital of Traditional Chinese Medicine, Shanghai Mental Health Center, Shanghai Pudong New District Nanhui Mental Health Center, and Shanghai Xuhui District Central Hospital. A total of 204 patients who meet the inclusion criteria will be recruited and randomly assigned into either group, receiving EA treatment with placebo drugs or ESC with sham acupuncture (SA) treatment. After a one-week baseline evaluation, participants will enter a nine-month observation period in this trial. Participants will receive the EA or SA treatment three times a week (every other day) for the first 8 weeks and twice a week (every two days) for the final 4 weeks. Participants will be assessed at the following time points: baseline (1 week before treatment), the 1st month of treatment (4 weeks after treatment starts), the 2nd month of treatment (8 weeks after treatment starts), the end of treatment (12 weeks after treatment starts), and follow-up (1 month, 3 months, and 6 months after treatment ends). All participants will complete the self-rated scales to assess their mental state and quality of life (see the detailed trial process in **Figures 1**, **2**). Throughout the trial, we will adhere to the Standards for Reporting Interventions in Clinical Trials of Acupuncture (STRICTA).

### Patients

#### Inclusion criteria

Participants meeting the following criteria will be included:

1. Participants of either gender, aged between 18 and 60 years.
2. Participants with psychiatrist-diagnosed depression based on the DSM-5 criteria (10).
3. Participants whose HAMD-17 score is between 17–24.
4. Participants who have not previously taken any type of antidepressant.
5. Participants who are assessed as having a low risk of suicide (i.e., score ≤ 2 in the Hamilton question of suicide).
6. Participants who have not received acupuncture treatment for at least 1 year.
7. Participants who are willing to participate in the trial and sign a written informed consent.

#### Exclusion criteria

Participants who indicate the presence of any of the following conditions will be excluded:

1. Participants with secondary depressive disorder caused by organic or psychotic disorders and other diseases.
2. Participants who are in the depressive episode of bipolar disorder or suffering from dysthymia, reactive depression, or depressive syndrome caused by other diseases.
3. Participants who had severe liver, kidney, heart, brain, or other organic diseases.
4. Participants with a history of alcohol abuse or drug dependence.
5. Pregnant or lactating women.

### Recruitment

The recruitment of participants will be conducted via the official websites of the centers, as well as through advertisements from outpatient clinics located within hospitals. Patients with depression who express interest in participating in the study are required to have a phone or online interview first. And then they will be asked to participate in face-to-face screening, during which they will be required to fill out forms with the guidance of psychologists or trained physicians. Before starting the intervention, participants who meet the eligibility criteria will be required to provide written informed consent.

### Sample size calculation

The non-inferiority hypothesis is based on the results of a previous study (11). HAMD-17, as the primary outcome, decreased by 11.3 points in the premedication group and by 10.2 points in the EA group, with an equal standard deviation of 11.5 points. Following these parameters, the proposed non-inferiority boundary is set at -4 points, with a level one type I statistical error equivalent to 0.025 and a desired statistical power standing at 80%. These calculations are supported by the analytical tools implemented in PASS 15.0. The results were obtained for a required sample size of 81 cases per group, and considering a 20% drop rate, 102 cases need to be included in each group, totaling 204 cases. The formula employed to determine the sample size in this trial was listed as:


$${\text{n}}_{\text{A}}={\text{k}}_{{\text{n}}_{\text{B}}}\;\text{and}\;{\text{n}}_{\text{B}}=\left(1+\frac{1}{\text{k}}\right){\left(\sigma \frac{Z_{1-\alpha }+Z_{1-\beta }}{\mu_{A}-\mu_{B}-\delta }\right)}^{2}$$

### Randomization and blinding

This study uses a single-blind design, concealing group allocations from participants. Only acupuncturists will be aware of the assignments, but they will not be involved in any other part of the trial, including enrollment, evaluation, and data analysis. To increase blind validity, treatment will be performed in a separate room. Meanwhile, communication about the treatment or efficacy between acupuncturists and participants will be avoided to the maximum extent. At the conclusion of the 12-week intervention, the Bang blinding index will confirm the effectiveness of the blinding technique (12). The placebo drugs supplied by Shanghai Xinyi Huanghe Pharmaceutical Co. Ltd. have an identical appearance to the proprietary form of ESC (Bailote), which is supplied by Sichuan Kelun Pharmaceutical Co. Ltd. All medications are regularly dispensed once a week, with seven tablets per box. Patients can pick up their medication from the dispenser at their own corresponding number on the pre-packaged envelopes. Before the start of the trial, each investigator will receive multiple training sessions on the implementation of the standards of this study and will strictly adhere to the principle of separation of departments.

The study will use a block randomization method with a block size of four, facilitated by SPSS version 24.0 software. Eligible participants will be randomly assigned to either the EA or ESC group in equal proportions. An independent researcher will enclose the treatment allocation codes in sequentially numbered opaque envelopes, and another independent researcher will assign participants to the groups based on the corresponding number on the pre-packaged envelopes and randomization schedule. To confirm the effectiveness of the blinding, the acupuncturist will inquire with all participants whether they received EA or SA treatment at the end of the trial. All participants’ information will remain confidential to the acupuncturist and assessors throughout the trial.

### Intervention

Treatment will last 12 weeks, with 3 sessions per week (usually every other day) for the first 8 weeks, decreasing to 2 per week (usually every two or three days) for the remaining 4 weeks, and the follow-up period will be 6 months. Each participant will be required to wear an eye patch and assume a supine position during each 25-minute session, which will be conducted in a private, tranquil environment. The treatment will be conducted by licensed acupuncturists with at least three years of experience in acupuncture practice. ESC and placebo tablets were identical in appearance and were administered as a morning dose. Participants will record details of the drug dose, duration of dosing, and response to dosing in a case report form (CRF). All drugs in the 7-dose kit will be dispensed once a week at regular intervals by specialized drug dispensers at each center. Patients may receive additional medication once a week if they need it, which will be recorded by the dispenser.

### EA group

Participants in the EA group will receive EA treatment with sterile disposable filiform needles (0.25×25 mm and 0.25×40 mm in size, Asia-med GmbH & Co. KG) and take the placebo drugs for three months. The main acupoints involve Baihui (GV20), Yintang (GV29), Guanyuan (RN4), Qihai (RN6), bilateral Zusanli (ST36), and bilateral Sanyinjiao (SP6). The matching points are Neiguan (PC6), Hegu (LI4), Taichong (LR3), Shenting (DU24), Fengchi (GB20), Shenmen (HT7), Sishencong (EX-HN1), Tianshu (ST25), Taixi (KI3), Xinshu (BL15), Ganshu (BL18), Pishu (BL20), Shenshu (BL23), and Shenmai (BL62). The acupuncturist will select two of the above matching points for each treatment according to the individual patient’s pattern. For the acupoints GV20, GV29, DU24, GB20, and EX-HN1, we used sterile, disposable filiform needles measuring 0.25×25 mm; whereas for the remaining primary acupoints, needles measuring 0.25×40 mm were employed. Following needle insertion, the acupuncturist will use rotating or lifting-thrusting techniques to induce the ‘De qi’ sensation. Three electrode pairs from the electro-stimulator (CMNS6-1, Wuxi Jiajian Medical Device Co., China) will be attached to the needles of GV20 and GV29, and bilateral ST36 and SP6 for 25 minutes, delivering a continuous wave with a frequency of 2 Hz, and the intensity will be set based on the tolerance of each patient. is composed entirely of starch, accounting for 100% of its content. The placebo, which consists entirely of starch (100% of its content), is administered simultaneously with EA. Patients are instructed to take the placebo orally once a day, at a dosage of 10 mg. Based on the individual patient’s response and clinical judgment, the dosage can be adjusted to 20 mg if necessary.

### ESC group

Participants will get SA treatment at the identical acupoints as those used in the EA group. The SA group will be administered using placebo needles (Streitberger Placebo needle, Asia-med GmbH & Co. KG) (13, 14). When the blunt needle tip touches the skin, patients will feel a pricking sensation, although no needle is inserted. An electro-stimulator will then be disposed of adjacent to the patients, and electrodes will be connected to the needles at the same acupoints as the EA group. The acupuncturist will activate the electro-stimulator with all settings at zero during the treatment. Patients will be notified when needles are extracted after 25 minutes of treatment. Dry cotton balls will then be applied to press at the acupoints so that patients can sense the removal of the ‘needles.’ We will use ESC as the participant’s antidepressant. The dose of ESC will be the minimum clinical starting dose of 10 mg once daily for three months. During treatment, patients were allowed to increase the dose (up to 20mg) according to the guidance of a professional psychiatrist.

### Outcome measurement

#### Primary outcome

The primary outcome will be the mean change in HAMD-17 score from baseline to week 12. The HAMD-17 is a questionnaire designed to evaluate the symptoms of individuals diagnosed with depression, which is an observer-rated questionnaire consisting of 17 items (15). The scale uses either a 3-point or 5-point rating system and has demonstrated strong reliability and validity, making it a widely used tool in both research and clinical settings. A higher overall score indicates a higher level of depression, with scores ranging from 0 to 54; scores of 0–6 indicate a normal person without depression; 7–16, likely or suspected to have depression; 17–24, mild to moderate depression; ≥25, severe depression. This outcome data will be collected at baseline, during the treatment phase (at 4, 8, and 12 weeks), and at the 16-, 24-, and 36-week follow-ups.

#### Secondary outcome

1. The depression recovery rate is the percentage of patients with a HAMD-17 score ≤7 after 12 weeks of treatment. The recovery rate refers to the percentage of individuals who have successfully overcome or managed their symptoms of depression, and will be collected at 12 weeks.
2. The depression remission rate is the percentage of patients between 8 ≤ HAMD-17 ≤ 12 after 12 weeks of treatment. The remission rate refers to the percentage of individuals who have achieved a significant reduction in symptoms of depression, which may involve some residual symptoms but not at levels that would meet the diagnostic criteria for depression. This outcome data will be collected at 12 weeks.
3. The Patient Health Questionnaire-9 (PHQ-9) is an essential self-administered depression screening and diagnostic tool for screening and assessing patients’ depressive status (16–18). It consists of nine items that are easily and effectively used to understand a subject’s depression in the past two weeks. The total scores might be anywhere from 0 (no depression symptoms at all) to 27 (daily occurrence of all symptoms). The higher the total score indicates, the more severe the depression. This outcome data will be collected at baseline, during the treatment phase (at 4, 8, and 12 weeks) and at the 16-, 24-, and 36-week follow-ups.
4. The 36-Item Short Form Health Survey (SF-36) is one of the most widely used quality-of-life assessment scales with good reliability and validity (19). It provides a comprehensive overview of the participants’ quality of life in eight dimensions: physiological functioning, physical functioning, somatic pain, general health status, energy, social functioning, emotional functioning, and mental health. Higher scores represent a better health status. This outcome data will be collected at baseline, and during the treatment phase (at 4, 8, and 12 weeks).
5. The dose and frequency of drugs will be recorded by participants based on actual daily dosing from baseline to the 6-month follow-up period.

### Adverse events

AEs include all detrimental or unintended indications, symptoms, or disorders derived from acupuncture therapy or the utilization of antidepressants, shall be managed following the agreed-upon protocol. The type and severity of all AEs should be diligently documented in the CRF. Needle pain, localized hemorrhage, palpitation, or dizziness both throughout and after the intervention are common AEs related to the EA treatment. Other prevalent AEs associated with drug treatment are migraines, nausea, abdominal bloating, and fatigue. If a severe adverse event (SAE) occurs, it should be reported to researchers and the Ethics Committee in detail within 24 hours of occurrence and assessed by the Treatment Emergent Symptom Scale (TESS) for further evaluation and management. Any subsequent recommendations issued by the Ethics Committee shall be conveyed to the Data Safety Monitoring Board (DSMB), which retains the authority to cease the trials at their discretion. The research team will actively monitor all recipients experiencing AEs until their resolution, particularly those participants who decide to withdraw owing to the occurrences.

### Statistical analysis

Analyses will be conducted on the intention-to-treat (ITT) population, which comprises all qualified participants who meet the inclusion criteria, have received the intervention at least once, and have provided at least one outcome assessment. In statistical analysis, any missing primary outcome data will be replaced by the last observed data point, in accordance with the ITT principle. Additionally, a Per-Protocol Set (PPS) analysis will be employed to assess those participants who completed the trial without significant protocol violations. Data analysis for this study will be performed using the statistical software SPSS V.24.0. Descriptive statistical analysis will be used for all demographic and clinical characteristics of subjects, such as sex, age, and weight. We will validate the homogeneity of demographic characteristics and examine the variables between the two groups. The number of patients who change their medication dosage will also be counted, and the variations between the two groups and within each group will be analyzed. If measurement data follows a normal distribution and has homogeneity of variance, the data values will be reported as mean ± standard deviation (MD ± SD). Covariance analysis will be used to compare the data across different groups. If the data does not adhere to the assumed normal distribution, the median values (P25, P75) will be used to characterize it. The Mann-Whitney U nonparametric test will be used to compare between groups. The mean ± standard deviation will be used for the description of the continuous variables. Qualitative data will be expressed in terms of frequency and percentage. The student’s t-test will be utilized for comparing measurement data between two groups from baseline to the 6-month follow-up, while the rank sum test will be used for ranked data, and the χ2 test will be used for analyzing categorical data. A paired t-test was used for comparisons before and after treatment within a group, and an analysis of variance was used for comparisons among different groups. Repeated-measurement analysis of variance was used to compare groups at multiple observation points. A significance level of 2.5% will be used for all statistical analyses, using two-tailed testing. Subgroup analysis will be conducted to examine differences between different genders and across various age brackets.

### Patient and public involvement

Patients with mild to moderate depression in the clinical department were consulted by the main researcher before the trial design. The treatment frequency and duration of this study were summarized from clinical experience and patients’ feedback. We will recruit all participants from the outpatient clinics in four centers. Patients who were involved in the consultation about the trial design before will not be recruited as participants. A journal article manuscript will be written to present the results after the trial completed, and a brief summary of results with plain language will be sent to all participants. The burden of intervention will not be assessed by participants themselves.

### Ethics and dissemination

All acupuncturists are licensed physicians with at least 3 years of experience in acupuncture treatment. They will participate in clinical training to ensure that EA and SA practices are standardized prior to the intervention.

To guarantee strict quality control during the implementation of the study and to ensure the reliability and reproducibility of the trial results, the project will establish a DSMB consisting of a professional in clinical research of acupuncture (Lixing Lao), a psychiatrist (Xiao Huang), and a statistician (Ruiping Wang). The DSMB will monitor whether the trial complies with the study design and standard guidelines or not. The DSMB will hold regular meetings to request progress reports from investigators and provide professional guidance and solutions to issues that may arise during the trial.

## Discussion

An epidemiological study of people with depression over a 12-month period found that 26.6%, 53.4%, and 20.0% were classified as having mild, moderate, and severe depression, respectively (20). Failure to promptly relieve the depressive state and its accompanying symptoms during early episodes of depression can contribute to its aggravation or recurrence, leading to impaired functioning, reduced productivity, and an increased risk of suicide, which can ultimately result in significant public health and economic consequences (21, 22). The question we put forward in this clinical trial is whether EA is not inferior to ESC for treating mild to moderate first-episode depression. ESC has high efficacy and favorable acceptability in patients with depression in China (23–25). By utilizing an established and effective SSRI as a positive control, we can more precisely evaluate the potential benefits and risks of the novel treatment (EA) in comparison to the standard-of-care therapy (ESC). However, the delayed onset of antidepressant action can prolong patients’ depressive feelings. AEs may also hinder therapeutic effects and result in high dropout rates, which leads to a large proportion of depressed patients who struggle to achieve complete remission and experience relapse. Thus, it is imperative to explore alternative or complementary treatments for depression, particularly in the early phase of the disease (26).

EA treatment can adjust the overall nervous, endocrine, and immune systems of the body across multiple levels, control the depressive emotions and somatization symptoms of patients with depression, and have the advantages of few side effects and long duration (27–29). Furthermore, EA offers a non-pharmacological alternative to traditional antidepressants, which could be particularly beneficial for patients who experience side effects or prefer non-drug treatments. Moreover, previous studies (9) have shown that EA treatment has a faster onset of action compared to SSRIs and functions as a safer complementary therapy. Nonetheless, the quality of evidence supporting the efficacy and safety of EA in treating depression remains at a low-to-moderate level. Most of these clinical trials had a high risk of bias, thus calling for rigorous randomized controlled trials. Besides, there are few clinical studies about EA treatment for the treatment of first-episode depression. The antidepressant effect of EA has not been previously proposed, but it may be associated with inhibiting hypothalamic-pituitary-adrenal (HPA) axis hyperactivity, regulating neuropeptides and neurotransmitters, decreasing proinflammatory cytokine levels, and restoring hippocampal synaptic plasticity, among other mechanisms (28).

Traditional Chinese medicine (TCM) theory states that depression is mainly caused by ‘qi’ stagnation, which subsequently leads to the disorder in the brain’s qi systems and an imbalance of yin and yang. In this trial, we will apply a semi-standard point selection consisting of core and additional acupoints, strictly following the syndrome differentiation of TCM theory and allowing some flexibility based on the individual patients’ specific symptoms. The Governor Vessel connects the brain directly and is commonly used to treat depressive disorders (30, 31). GV20 and GV29 are often used to promote the recovery of depressive disorder, as they may facilitate the flow of qi and blood in the brain and restore brain function by ameliorating cognitive deficits (28, 32). The Ren Meridian, has the same origin as the Governor Vessel Meridian. RN6 and RN4, together with ST36 and SP6, can supplement the deficiency and drain the excess, tonifying Qi and blood and relaxing the patient (33), thus regulating the somatization symptoms in conjunction with the Governor Vessel as a whole. In addition, based on the clinical experience of many professional acupuncturists and our previous clinical studies (29, 34), the acupoints we applied effectively improved poor minds and relieved depressive symptoms. The long-range effect is also an important factor of acupuncture treatment, and thus we set long enough intervention period and follow-up period. It will be possible to investigate the enduring effects of acupuncture on depression and ascertain the duration of the therapeutic effect. However, there are still several challenges and limitations in this study. Firstly, it is not feasible to achieve blinding of the acupuncturists during the intervention period, and thus rigorous training will be provided to minimize any potential bias. Acupuncturists will not be involved in either assessing patients or the analysis. Secondly, we are concerned that the initial clinical dose of ESC (10mg) was not effective in relieving depressive symptoms in some patients and therefore allowed them to increase the dose (up to 20 mg) according to their symptoms under the guidance of their psychiatrists. Increasing the drug dose may provide more effective clinical benefits to patients, but at the same time, we cannot be sure whether the changes brought about by the dose of the drug are due to purely time effects or a cumulative dose effect. Thirdly, the outcome of this study was subjective and vulnerable to reporting bias. Further studies should focus on precise objective outcome assessment. Finally, we set a 12-week follow-up time, which may lead to an increased dropout rate. Therefore, more flexible approaches should be employed during the follow-up period, such as making online inquiries or evaluating by phone.

## Conclusion

In summary, we will strictly adhere to the CONSORT statement and STRICTA recommendations to ensure standardized acupoint selection, manipulation, evaluation, and therapist clinical experience. As a multi-center RCT in a first-line city, we expect that this trial will validate the effectiveness and safety of EA as a clinical intervention for mild to moderate first-episode depression, which will address the gap left by SSRIs and provide a low-risk, beneficial non-drug therapy for patients with depression.
